# Mitochondrial genome variability and metabolic alterations reveal new biomarkers of resistance in testicular germ cell tumors

**DOI:** 10.20517/cdr.2024.141

**Published:** 2024-12-18

**Authors:** Pavlina Kabelikova, Danica Ivovic, Zuzana Sumbalova, Miloslav Karhanek, Lucia Tatayova, Martina Skopkova, Michal Cagalinec, Vladimira Bruderova, Jan Roska, Dana Jurkovicova

**Affiliations:** ^1^Cancer Research Institute, Biomedical Research Center, Slovak Academy of Sciences, Bratislava 84505, Slovak Republic.; ^2^Institute of Medical Chemistry, Biochemistry and Clinical Biochemistry, Faculty of Medicine, Comenius University in Bratislava, Bratislava 81108, Slovak Republic.; ^3^Institute of Experimental Endocrinology, Biomedical Research Center, Slovak Academy of Sciences, Bratislava 84505, Slovak Republic.; ^4^Department of Medical Genetics, Medirex Inc., Bratislava 82104, Slovak Republic.; ^#^Co-senior authors.

**Keywords:** Testicular cancer, chemoresistance, mitochondria, mtDNA mutation, OXPHOS, glycolysis

## Abstract

**Aim:** Mutations in the mitochondrial (mt) genome contribute to metabolic dysfunction and their accumulation relates to disease progression and resistance development in cancer cells. This study explores the mutational status of the mt genome of cisplatin-resistant *vs.* -sensitive testicular germ cell tumor (TGCT) cells and explores its association with their respiration parameters, expression of respiratory genes, and preferences for metabolic pathways to reveal new markers of therapy resistance in TGCTs.

**Methods:** Using Illumina sequencing with Twist Enrichment Panel, the mutations of mt genomes of sensitive 2102EP, H12.1, NTERA-2, T-cam and resistant 2102EP Cis, H12.1 ODM, 1411HP, 1777NRpmet, NTERA-2 Cis and T-cam Cis cell lines were identified. The mt respiration of the cells was assessed using high-resolution respirometry method (O2k-respirometer Oroboros) and the differential expression profiles of mt respiratory genes were determined using RT-qPCR. Associated preferences for metabolic pathways were compared using Glycolysis/OXPHOS assay.

**Results:** In resistant TGCT cells, new mutations in mt genes *MT-ND1-6, MT-RNR, MT-CO1-3, MT-ATP6*, and *MT-CYB* were recognized. The respiratory rates of the 1777NRpmet cell line were the highest, while those of the 1411HP line the lowest; rates of the control and all other TGCT cell lines fell between these two lines. The statistically significant differences in gene expression of the respiratory genes were recorded only in NTERA-2 Cis and T-cam Cis cell lines. Sensitive cell lines NTERA-2 and 2102EP preferred oxidative phosphorylation (OXPHOS), while glycolysis was typical for resistant NTERA-2 Cis, 2102EP Cis and 1411HP cell lines. Metastatic 1777NRpmet cells seem to utilize both. An isogenic pair of cell lines H12.1 and H12.1ODM showed the opposite dependence, sensitive H12.1 preferring glycolysis, while resistant H12.1ODM OXPHOS.

**Conclusion:** In summary, our study identified new mutations in mt genes of resistant TGCT cell lines that are associated with different mt respiration parameters, gene expression patterns and preferences for metabolic pathways, providing potential novel molecular biomarkers that distinguish the resistant TGCT phenotype or specify its histological classification.

## INTRODUCTION

Testicular germ cell tumors (TGCTs) are relatively rare tumors, but the most frequently occurring solid malignancies in young men. After the introduction of cisplatin (CDDP) to chemotherapy regimens, TGCTs have become highly curable, with an efficacy rate of up to 95%^[1]^. However, despite the high success rate of this treatment, some patients develop CDDP resistance, resulting in poor prognosis^[2]^. In general, TGCTs exhibit a relatively low number of somatic mutations, but recent advances in sequencing technologies have identified several risk loci specific to this malignancy. Genetic alterations in tumor cells play an important role during neoplastic progression and in the treatment response^[3]^. Changes and mutations in genes, germline or somatic, can affect their proteins and signaling pathways, ultimately contributing to the development or progression of the disease. The lack of known driver mutations in TGCTs suggests that key aspects of tumorigenicity and chemotherapy response in this malignancy may rather be under epigenetic control^[4]^.

Therapeutic resistance of malignant cells is often associated with defective apoptosis. The successful elimination of tumor cells is, therefore, highly dependent on the ability of treatment to stimulate suppressed apoptotic pathways, making mitochondria a promising target for such an approach^[5]^. Mitochondria, which probably evolved as a proteobacterium engulfed by a pre-eukaryotic cell, are organelles with their own DNA encoding proteins that regulate numerous metabolic processes and signaling pathways, potentially playing a major role in cancer and therapy response^[6]^. In eukaryotic cells, mitochondria are metabolic centers producing adenosine triphosphate (ATP) for the energy supply for cellular functions^[7]^. To maintain these functions, mitochondria rely on the genetic information stored in the nucleus and their own small genome (mtDNA/mt genome). Mammalian cells have numerous copies of mtDNA, a 16.6 kilobase (kb) circular, double-stranded molecule encoding 13 proteins essential for electron transport (ET) and ATP synthesis, 22 transfer RNAs, and 2 ribosomal RNAs^[8]^.

Metabolic dysfunction is typical for malignant cells, and mutations of the mt genome may contribute to the metabolic deficit^[9]^. CDDP is known to accumulate not only in the nucleus but also in the mitochondria. It forms adducts with mtDNA and proteins, deregulating their functions. Mitochondria ensure the production of ATP through oxidative phosphorylation (OXPHOS). The enzymes involved in OXPHOS are multisubunit complexes encoded by both nuclear and mtDNA. Regulation of respiration is thus a highly coordinated process, which ensures that the production, assembly, and function of mitochondria meet the energy needs of the cell^[10]^.

Mitochondria are also the key source of reactive oxygen species (ROS). Alongside the cytostatic effect of the chemotherapy, increased ROS contribute to the induction of oxidative stress that triggers mitochondria-mediated cell death in the treatment of tumors, including TGCTs. However, in tumor cells, mitochondria can be damaged, but survive and accumulate a number of mutations. The accumulation of these mutations leads to disease progression, advancement of the stages of the disease, and development of chemoresistance^[11]^. To reveal the causes of chemoresistance, it is therefore important to identify genetic changes in both nucleus and mitochondria, as well as the principles of their mutual communication. Understanding the complex role of mitochondria in cancer development and therapeutic response may reveal new therapeutic targets and potential biomarkers for CDDP-resistant TGCT patients.

In our work, we focused on the identification of specific changes in the mt genomes (new mutations and differential gene expression) that could potentially distinguish CDDP-sensitive and -resistant TGCT cells. We associated these changes with physiological parameters – respiration, assessed by measuring their O_2_ consumption, and metabolic pathway dependence, assessed by determining the metabolic preferences or shift toward OXPHOS or glycolysis, used for ATP production. With this approach, we aimed to identify the genetic variations of mt genomes in TGCTs that would represent markers of changed mitochondrial function potentially contributing to chemoresistance and tumor aggressiveness. Accordingly, new potential biomarkers for the detection of chemoresistance in TGCTs were revealed.

## METHODS

### Cell cultures

In our analyses, we used 10 TGCT cell lines with a different response to CDDP and a degree of intrinsic and acquired resistance to CDDP (kindly provided by Dr. Thomas Mueller, Martin Luther University Halle-Wittenberg, Halle, Germany and Dr. Katarina Kalavska, National Cancer Institute, Bratislava, Slovakia): sensitive - 2102EP, H12.1, NTERA-2, T-cam, resistant - 2102EP Cis, H12.1 ODM (alternatively H12.1D), 1411HP, 1777NRpmet, NTERA-2 Cis, T-cam Cis, and control cell line Hs1. Tes (ATCC, CRL-7002) used as a healthy reference in our analyses. Characteristics of most of these cell lines can be found in Roška *et al*. 2020^[12]^. Therapy resistance is typical for non-seminomatous TGCTs, while seminomas respond to CDDP treatment very well. Accordingly, we preferentially used non-seminomatous TGCT cell lines and their resistant derivatives or counterparts. To identify potential differences, we have also used seminomatous sensitive TGCT cell line T-cam and its resistant derivative T-cam Cis. The resistant cell line H12.1 ODM is an isogenic derivative of the cell line H12.1 created by cultivation in differentiation-inducing media^[13]^. Lines 1411HP and 1777NRpmet were derived from metastases of TGCT patients who did not respond to any treatment, and therefore were considered intrinsically resistant to CDDP. The NTERA-2 Cis and T-cam Cis cell lines are isogenic derivatives of the sensitive parental cell lines; their resistance to CDDP was acquired by cultivation in increasing CDDP concentrations in culture media. The H12.1, H12.1ODM, 1411HP, 1777NRpmet, T-cam, and T-cam Cis TGCT cell lines were grown in RPMI-1640 medium supplemented with 10% fetal bovine serum (FBS), penicillin (100 units/mL), and streptomycin (10 µL/mL). The NTERA-2, NTERA-2 Cis and 2102EP, 2102EP Cis, and Hs1. Tes TGCT cell lines were grown in Dulbecco’s modified Eagle’s medium (DMEM) with F-10 nutrient mixture (1:1), 10% FBS, penicillin (100 units/mL), and streptomycin (10 µL/mL). Cell lines were cultivated at 37 °C in 5% CO_2_.

### RNA extraction

To extract the total RNA from cells, TRI Reagent solution (Life Technologies, Frederick, MD, USA) was used. The total RNA quantification and purity were assessed using NanoDropTM Lite Spectrophotometer (Cat. ND-LITE, Thermo ScientificTM, Waltham, MA, USA) and Qubit fluorometer (Life Technologies, Frederick, MD, USA). RNA integrity was assessed using the Agilent 2100 Bioanalyzer (Agilent, San Diego, CA, USA). Extracted RNA was used for mt genome sequencing and RT-qPCR analysis.

### Next-generation sequencing

Using the TWIST panel (Twist BIOSCIENCE, San Francisco, CA, USA), we determined the mutation profile of the mt genome of resistant TGCT cell lines (1777NRpmet, 1411HP, 2102EP Cis, H12.1 ODM, T-cam Cis) compared to sensitive TGCT cell lines (2102EP, H12.1, T-cam). A library was prepared using Twist Library Preparation Kit (# 104384, Twist BIOSCIENCE, San Francisco, CA, USA) and Twist Enrichment Panel Kit (# 102038, Twist BIOSCIENCE, San Francisco, CA, USA). Samples were sequenced using the Illumina iSeq system (Illumina, Santa Clara, CA, USA). Data were analyzed using Illumina software, validated in IGV and MITOMAP, and initial bioinformatics and statistical analysis were performed.

### Bioinformatics

For the calculation of mutation frequency (number of mutations per base pair), we used a calculation as previously described by Balin and Cascalho^[14]^. It is assumed that each mutation reflects an independent event that has a small probability of occurrence and has an infinitesimal probability of occurring twice at the same position. Thus, each base pair could be considered an independent Bernoulli trial with an output of either mutated or not mutated and the average number of mutations in a gene could be calculated using the zero order of the Poisson process. The average frequency of mutations (N0/N, where N0 is the number of unaltered positions and N the total number of bases sequenced) thus equals e^-λ and from this λ = -ln (N0/N), where λ represents the average number of mutations per base pair, i.e., frequency of mutation.

### Mitochondrial respiration

**Figure 1:**
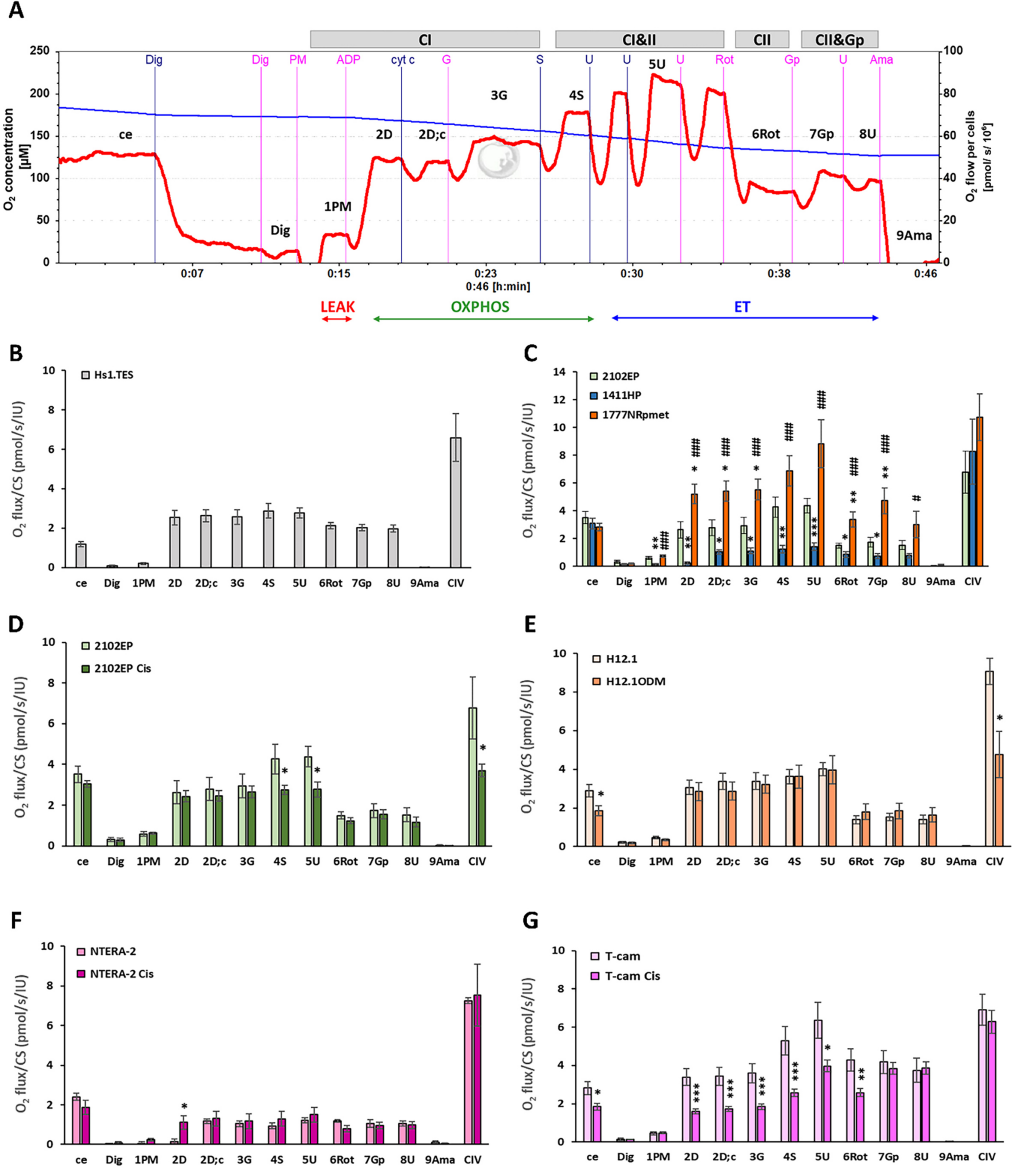
Mitochondrial respiration in TGCTs resistant and sensitive cell lines. (A) The representative trace from the measurement of mitochondrial respiration following the SUIT-008 protocol^[16]^. The blue line represents the concentration of O_2_ in the chamber and the red line shows the consumption of O_2_ as flow per cells (pmol/s/10^6^ cells). A total of 1 × 10^6^ cells was used in a 2 mL chamber of an O2k-respirometer and mitochondrial respiration of the TGCT cells was measured in mitochondrial respiration medium MiR05^[15]^ with 20 mM creatine at 37 °C under continuous stirring at 750 rpm. The trace is from the measurement of the 2102EP cell line. After stabilization at ce, cells were permeabilized by Dig and chemicals were consecutively added to the O2k chamber following SUIT-008 protocol: PM; ADP; cyt c; G; S; U; Rot; Gp; U; Ama. All substrates were titrated in kinetically saturating concentrations and uncoupler FCCP was titrated in optimum concentration to reach the maximum flux. The descriptions above and below the graph show: CI: Complex I pathway; CI&II: complex I and II pathways; CII: complex II pathway; CII&Gp: complex II and GpDH pathways; LEAK: non-phosphorylating resting state of respiration; OXPHOS: the phosphorylating state of respiration; ET: noncoupled state of respiration at optimum uncoupler concentration. The labels above the red line assign the respiration after the titration steps. (B-G) Parameters of mitochondrial respiration in cell lines measured following SUIT protocol 008^[16]^ evaluated as O_2_ flux/CS (pmol/s/IU). The columns represent the mean ± SEM of the respiratory capacities after the titration steps shown in Figure 1A. For evaluation of CIV activity, artificial substrates TMPD and ascorbate were titrated after Ama supporting electron flow to CIV, then sodium azide (200 mM) was added to inhibit CIV. The activity of CIV was evaluated as a difference between O_2_ fluxes before and after the addition of sodium azide^[16]^. 1411HP, 1777NRpmet: intrinsically resistant TGCT cell lines; T-cam: T-cam Cis - seminomas TGCT cell lines; H12.1/H12.1ODM, 2102EP/ 2102EP Cis, NTERA-2/ NTERA-2 Cis: non-seminomatous TGCT cell line pairs (sensitive/resistant); Hs1.Tes: control cell line. ^*^P ≤ 0.05, ^**^P ≤ 0.01, ^***^P ≤ 0.001 for resistant *vs*. sensitive variant of the cell line; ^#^*P* ≤ 0.05, ^###^*P* ≤ 0.001 for 1777NRpmet *vs*. 1411HP line. TGCT: Testicular germ cell tumors; ce: routine respiration; Dig: digitonin; PM: pyruvate + malate; ADP: adenosine diphosphate; cyt c: cytochrome c; G: glutamate; S: succinate; U: uncoupler; Rot: rotenone; Gp: glycerophosphate; U: uncoupler; Ama: antimycin A; FCCP: carbonyl cyanide-p-trifluoromethoxyphenylhydrazone; CIV: complex IV.

Mitochondrial respiration was measured with high-resolution respirometry method^[15]^ using O2k-respirometer (Oroboros Instruments, Austria). For respirometric analysis, we used 1 × 10^6^ cells, which were trypsinized, spun, and resuspended in PBS before measurement. Subsequently, the cells were added into the oxygraph chamber with mitochondrial respiration medium MiR05 with 20 mM creatine at 37 °C under continuous stirring at 750 rpm. The individual substrates, uncouplers, and inhibitors were added in the sequence following substrate-uncoupler-inhibitor titration (SUIT)-008 protocol^[15,16]^: digitonin (Dig) (20-60 µg/10^6^ cells), pyruvate (P) (5 mM) + malate (M) (2mM), ADP-Mg (2.5 mM, 0.6 mol MgCl_2_/mol ADP), cytochrome c (cyt c) (10 µM), glutamate (G) (10 mM), succinate (S) (10 mM), uncoupler carbonyl cyanide p-trifluoro-methoxyphenyl hydrazone (FCCP, U) (0.25 µM steps), complex I (CI) inhibitor rotenone (Rot) (0.5 µM), glycerophosphate (Gp) (10 mM), uncoupler FCCP (U) (0.5 µM steps), and antimycin A (Ama) (2.5 µM). The schematic representation of SUIT- 008 protocol is presented in Figure 1A. For evaluation of complex IV (CIV) activity, N,N,N´,N´-Tetramethyl-p-phenylenediamine dihydrochloride (TMPD, 0.5 mM) + ascorbate (2 mM) were added after Ama, followed by the inhibitor of CIV sodium azide (200 mM). The respiration after Ama (or after Dig if lower) representing residual oxygen consumption was subtracted from all respiratory rates.

### Citrate synthase activity determination

The activity of citrate syntyhase (CS), as a marker of mitochondria, was determined spectrophotometrically as described by Eigentler *et al*.^[17]^.

### RT-qPCR

Using RevertAid First Strand cDNA Synthesis Kit (Thermo Scientific, Waltham, MA, USA), cDNA was synthesized from the isolated total RNA samples. Afterwards, the relative expression of respiratory genes corresponding to their cognate subunits of the ET chain: *MT-ND1-6* (CI), *MT-CYB* (complex III), *MT-CO1-3* (CIV*)* and *MT-ATP6, MT-ATP8* [complex V (CV)] and of housekeeper gene *B-actin* were determined using SYBR Premix Ex Taq II (Tli RNaseH Plus, Takara, Japan) and compared between resistant TGCT cell lines (H12.1 ODM, 1411HP, 1777NRpmet, T-cam Cis, NTERA-2 Cis) and sensitive ones (H12.1, 2102EP, NTERA-2, and control line Hs1.Tes). Oligonucleotides used for qPCR analyses are listed in Table 1.

**Table 1:** Oligonucleotides used for qRT-PCR analyses

**GENE**		**Sequence (5’-3’) - FW**	**Sequence (5’-3’) - REV**
*MT-ND1*	NADH dehydrogenase subunit I	CGATTCCGCTACGACCAACT	AGGTTTGAGGGGGAATGCTG
*MT-ND2*	NADH dehydrogenase subunit II	ACCATCTTTGCAGGCACACT	GCTTCTGTGGAACGAGGGTT
*MT-ND3*	NADH dehydrogenase subunit III	GCGGCTTCGACCCTATATCC	AGGGCTCATGGTAGGGGTAA
*MT-ND4*	NADH dehydrogenase subunit IV	GCTTCACACCTCATATCCTCCC	GCTAAGAGGGAGTGGGTGTT
*MT-ND5*	NADH dehydrogenase subunit V	CTAGCAGCAGCAGGCAAATC	GTGCTTGAGTGGAGTAGGGC
*MT-ND6*	NADH dehydrogenase subunit VI	TCCCCCGACAATCTCAATTAC	GGTCAGGGTTGATTCGGGAG
*MT-CYB*	Cytochrome b	CCCCACCCCATCCAACAT	TCAGGCAGGCGCCAAG
*MT-CO1*	Cytochrome c oxidase subunit I	AGCCTCCGTAGACCTAACAA	CGAAGCGGGGCGTTTGGTAT
*MT-CO2*	Cytochrome c oxidase subunit II	ACTTTCACCGCTACACGACC	GGCATGAAACTGTGGTTTGCT
*MT-CO3*	Cytochrome c oxidase subunit III	ACATACCAAGGCCACCACAC	AGGCTCAGAAAAATCCTGCGA
*MT-ATP6*	ATP synthase 6	CCAATAGCCCTGGCCGTAC	CGCTTCCAATTAGGTGCATGA
*MT-ATP8*	ATP synthase 8	ACTACCACCTACCTCCCTCAC	GGATTGTGGGGGCAATGAATG
*B-actin*	Housekeeper	GCACTCTTCCAGCCTTCCTT	CGTACAGGTCTTTGCGGATG

qRT-PCR: Quantitative reverse transcription with polymerase chain reaction; FW: forward; REV: reverse; NADH: nicotinamide adenine dinucleotide.

### Mitochondrial metabolism

To analyze the metabolic pathway dependence of individual cell lines, we used the Glycolysis/OXPHOS Assay Kit (# G270, Dojindo, Laboratories, JAPAN) following the manufacturers’instructions.

### Statistical analysis

Data of relative expression are presented as the mean relative expression (RE, 2^-ΔCt^) of TGCT cell lines from three technical and three biological replicates. Data were normalized using the housekeeper gene B-actin. Error bars represent SD. In the case of respiration, oxygen flux was normalized to mitochondrial marker CS activity for each TGCT cell line. Data are presented as mean O_2_ flux/CS ± SEM from four to six biological replicates. Statistical significance: ^*^P ≤ 0.05, ^**^P ≤ 0.01, ^***^P ≤ 0.001. The difference in relative ATP amount and lactate production in the analysis of the metabolic pathway dependence was determined by One-way ANOVA with Bonferroni (if normally distributed and homoscedastic) and/or Tamhane’s T2 (if normally distributed and heteroscedastic) post-hoc tests for multiple comparison.

## RESULTS

### Determination of mtDNA mutational status in TGCT cell lines

Using next-generation sequencing (NGS) approach, we determined the mutational profile and identified genetic changes of the mt genome of the individual resistant TGCT cell lines (1777NRpmet, 1411HP, 2102EP Cis, H12.1 ODM, T-cam Cis) in comparison with sensitive TGCT cell lines (2102EP, H12.1, T-cam). Most of the identified mutations were located in the genes encoding the subunits of the respiratory chain proteins. Some mutations (mostly single nucleotide polymorphisms - SNPs) have already been described, but our analysis also revealed new, yet undescribed mutations in these cell lines. Tables 2 and 3 show the identified changes and their positions.

**Table 2:** Genetic variability identified by comparing the resistant *vs*. sensitive TGCT cell lines

**Sensitive cell line**	**Resistant cell line**	**Position**	**REF**	**ALT**	**RS_ID**	**anno GENE**	**AMK change**	**APOGEE2 prediction (for missense)**	**MitoTIP (%)**	**Heteroplasmy (%)**	**gnomAD (%)**
2102EP	2102EP Cis	4839	C	A	.	*MT-ND2*	L124M	↓↓0.134		38.8	0
	1411HP	8856	G	A	rs878853078	*MT-ATP6*	A110A				0.6
		10211	C	T	.	*MT-ND3*	F51F				0.131
		10389	T	C	.	*MT-ND3*	L111L			47.0	0.05
		12360	C	T	.	*MT-ND5*	T8T				0.41
		12425	-	A	.	*MT-ND5*	frameshift			16.0	0
	1777NRpmet	1542	T	C	.	*MT-RNR1*					0.009
		3086	T	C	.	*MT-RNR2*					0
		6931	G	A	.	*MT-CO1*	G343E	↑0.68		18.8	0
		9812	C	T	.	*MT-CO3*	G202G				0.014
H12.1	H12.1 ODM	3481	G	A	rs587776433	*MT-ND1*	E59K	↑↑0.893		18.0	0
		5839	C	T	.	*MT-TY*			↓↓5.50	13.0	0.082
		8243	G	A	.	*MT-CO2*	E220K	↓0.295			0
		12389	C	T	.	*MT-ND5*	P18L	↓0.258			0.007
		15215	G	A	.	*MT-CYB*	G157Ter			5.2	0
		15797	G	A	.	*MT-CYB*	G351Ter			7.4	0
		16304	T	C	rs386829305	*MT-CR*					5.5
	1411HP	3902	ACCTT	AAGGT	.	*MT-ND1*	DLA199GKV			4.0	NR
		10211	C	T	.	*MT-ND3*	F51F				0. 131
		10389	T	C	.	*MT-ND3*	L111L			47.0	0.051
		12360	C	T	.	*MT-ND5*	T8T				0.041
		12425	-	A/AA	.	*MT-ND5*	frameshift			16.0	0
	1777NRpmet	309	C	CC	rs878871521	*MT-CR*				4.5	0.058
		1542	T	C	.	*MT-RNR1*					0.009
		3086	T	C	.	*MT-RNR2*					0
		6931	G	A	.	*MT-CO1*	G343E	↑0.68		18.8	0
		9812	C	T	.	*MT-CO3*	G202G				0.014
T - cam	T - cam Cis	10237	T	C	rs193302927	*MT-ND3*	I60T	↑0.666			0.179
		12425	A	-	.	*MT-ND5*	frameshift			1.7	0

For bioinformatics analysis, mutations in the resistant cell line were always compared against the corresponding sensitive counterpart. ↓: possibly benign; ↓↓: likely benign; ↑: possibly pathogenic; ↑↑: likely pathogenic. The mutations in the table represent mutations found in resistant TGCT cell lines - 2102EP Cis, 1411HP, 1777NRpmet compared to the sensitive 2102EP cell line, and H12.1 ODM, 1411HP, 1777NRpmet compared to H12.1 cell line. A T-cam Cis was compared against the parental T-cam only, as both represent a seminomatous TGCT cell line. REF: reference allele; ALT: alternative allele detected; rs_id: ID of known polymorphism; dot/“.”: unknown polymorphism; anno GENE: annotation of the gene where polymorphism was detected; NR: no record in the database. Variants in bold are confirmed likely pathogenic or pathogenic variants when found in the germline (source Mitomap). The level of heteroplasmy is stated in variants found in the heteroplasmic state; otherwise, the variants were homoplasmic. TGCT: Testicular germ cell tumors.

**Table 3:** Genetic variability identified by comparing the resistant TGCT cell lines to the rCRS

	**Resistant cell line**	**Position**	**REF**	**ALT**	**RS_ID**	**anno Gene**	**AMK change**	**APOGEE2 prediction (for missense)**	**MitoTIP (%)**	**Heteroplasmy (%)**	**gnomAD (%)**
rCRS	1777NRpmet	10464	T	C	rs1556423812	*MT-TR*			↑51.5		0
		10874	C	T	rs1556423869	*MT-ND4*	L39L				0.021
		11252	A	G	rs879229170	*MT-ND4*	I165V	↓↓0.023			0.087
		15933	G	A	.	*MT-TT*			↑66.8		NR
		16164	A	G	rs879051423	*MT-CR*					0.09
		16173	C	T	rs1556424780	*MT-CR*					0.094
	1411HP	8857	G	A	rs201017581	*MT-ATP6*	G111S	↓↓0.024			0.039
		10874	C	T	rs1556423869	*MT-ND4*	L39L				0.021
		14767	T	C	rs1603224866	*MT-CYB*	T7T				0.05
		15933	G	A	.	*MT-TT*			↑66.8		NR
		16173	C	T	rs1556424780	*MT-CR*					0.094
		16191	C	T	rs1556424795	*MT-CR*				2.2	NR

Metastatic resistant TGCT cell lines, 1777NRpmet and 1411HP, were also compared against the reference genome, as they do not have their isogenic sensitive counterpart. ↓↓: likely benign; ↑: possibly pathogenic. Heteroplasmy level is stated in variants found in heteroplasmic state; otherwise, the variants were homoplasmic. REF: Reference allele; ALT: alternative allele detected; rs_id: ID of known polymorphism; dot/“.”: unknown polymorphism; anno GENE: annotation of the gene where polymorphism was detected; NR: no record in the database. TGCT: Testicular germ cell tumors; rCRS: mitochondrial reference genome.

As the metastatic TGCT cell lines 1411HP and 1777NRpmet do not have a sensitive counterpart, we compared these lines against the reference mt genome (rCRS) [Table 3]. This comparison identified three homoplasmic variants as potentially pathological. MitoTIP, an in silico tool for predicting the pathogenicity of novel mitochondrial tRNA variants, showed that the homoplasmic variant 10464T > C in *MT-TR* has already been described as likely pathogenic with a value of 0.515. Additionally, the newly identified homoplasmic variant 15933G > A, identified in both metastatic TGCT lines, has been specified as likely pathogenic with a value of 0.668 and is associated with mitochondrial disease. Unfortunately, the mutation status of the mt genome of NTERA-2 /NTERA-2 Cis and Hs1.Tes cell lines was not analyzed because they did not meet sufficient quality criteria in the process of preparing the sequencing library.

Comparing the resistant cell line 1777NRpmet with two sensitive cell lines 2102EP and H12.1, we discovered new mutations that are not present in any of the sensitive cell lines. The newly identified homoplasmic variant 1542T > C was located in the *MT-RNR1*, 3086T > C in *MT-RNR2*, and heteroplasmic variant 6931G > A located in *MT-CO1* and 9812C > T in *MT-CO3* genes. In the second metastatic line, 1411HP, we identified new, so far undescribed homoplasmic mutation 10211C > T and heteroplasmic variant 10389T > C located in *MT- ND3*, homoplasmic 12360C > T and heteroplasmic 12417 - > A variants in *MT-ND5* genes. Next, we revealed an inversion of seven nucleotides, located between positions 3902 and 3908 in *MT-ND1*. Metastatic 1411HP cell line was the only one displaying mutation in the *MT-ATP6* gene (8856G > A), CV of the respiratory chain, important for ATP production. In the case of TGCT cell line 2102EP Cis, one new mutation 4839C > A in the *MT-ND2* gene was identified with a high level of heteroplasmy, compared to its sensitive counterpart 2102EP. In TGCT cell line H12.1 ODM, newly identified heteroplasmic mutation 5839C > T was located in the *MT-TY*, and homoplasmic variants 8243G > A in *MT-CO2*, 12389C > T in *MT-ND5*, and 15215G > A and 15797G > A in *MT-CYB* genes. The NGS analysis identified one new mutation 12425A > - in the *MT-ND5* gene in the seminomatous T-cam Cis TGCT cell line. The mutation frequency of all individual mitochondrial genes across analyzed TGCT cell lines was determined using the Mutect2 tool [Table 4].

**Table 4:** Mutation frequency of mitochondrial genes in TGCT cells

**Gene**	**Exonic mutation frequency**
*MT-TP*	1.5826 × 10^-2^
*MT-TR*	2.4649 × 10^-3^
*MT-TQ*	1.3864 × 10^-3^
*MT-RNR1*	1.2418 × 10^-3^
*MT-ND3*	1.0270 × 10^-3^
*MT-CYB*	6.1036 × 10^-4^
*MT-ND5*	4.4013 × 10^-4^
*MT-ND1*	4.1080 × 10^-4^
*MT-ATP6*	3.8621 × 10^-4^
*MT-RNR2*	3.4788 × 10^-4^
*MT-ND2*	2.8949 × 10^-4^
*MT-ND4*	2.7392 × 10^-4^
*MT-CO3*	2.7197 × 10^-4^
*MT-CO1*	2.2067 × 10^-4^
*MT-CO2*	1.6304 × 10^-4^

Only recurrent frequencies of mutations observed in all resistant TGCT cell lines with the highest score are presented. The highest exonic mutation frequency was observed in the *MT-TP* gene encoding the amino acid proline, *MT-TQ* encoding the amino acid glutamine, *MT-TR* encoding the amino acid arginine, followed by *MT-RNR1*, complexes I, III, IV, and V respiratory, and the lowest mutation frequency is *MT-COII*. *MT-TP*: tRNA Pro; *MT-TR*: tRNA Arg; *MT-TQ*: tRNA Gln; *MT-RNR1/2*: 12S rRNA; TGCT: testicular germ cell tumors.

### Mitochondrial respiration analysis

Since most of the identified mt genome mutations were located in the genes encoding components of the respiratory chain, we further explored the potential functional consequences and compared the respiration activity of selected TGCT cell lines using high-resolution respirometry method^[15]^. The analysis of the function of the respiratory chain revealed significant differences between resistant and sensitive cell lines, as well as between those exhibiting resistant phenotypes. Noticeably, the 1777NRpmet-resistant TGCT cell line showed the highest O_2_ consumption after the addition of individual substrates. In more detail, the respirometric analysis of different CDDP-resistant versus -sensitive TGCT cell lines by SUIT-008 protocol^[15,16]^ [Figure 1] showed significant changes in resistant 1411HP and 1777NRpmet cell lines, where CI-linked OXPHOS capacity (after ADP addition - the respiration associated with ATP production) was significantly decreased in 1411HP and significantly increased in 1777NRpmet compared to healthy control Hs.1 Tes and sensitive 2102EP cell lines.

The respiratory pattern of the control cell line Hs1.Tes is shown in Figure 1B and that of two intrinsically resistant TGCT cell lines, 1411HP and 1777NRpmet, compared to the sensitive 2102EP cell line are shown in Figure 1C. The respiratory rates of the 1777NRpmet cell line were the highest, while those of the 1411HP line the lowest. The respiratory rates of the control and all other TGCT cell lines fell between these two lines. The mitochondria of cell lines 1777NRpmet, T-cam/ T-cam Cis, H12.1/ H12.1 ODM, 2102EP/ 2102EP Cis respiring with CI-linked substrates P and M responded to addition of ADP by increasing respiration. The respiration also increased after the addition of CII-linked substrate S and after uncoupler, and decreased after inhibition of CI by Rot. Addition of Gp stimulated O_2_ consumption only in lines 1777NRpmet and T-cam Cis [Figure 1C and G], suggesting the importance of this GpDH-linked pathway in these cell lines. On the other hand, lines 1411HP and NTERA-2 did not respond to ADP. The respiration increased slightly after the addition of cyt c, suggesting impaired integrity of the outer mitochondrial membrane (OMM). There was almost no further response to any of the added substrate or uncoupler. CI inhibitor caused a slight decline in respiration in the 1411HP cell line, and no response in the NTERA-2 cell line. On the contrary, in the NTERA-2 Cis cell line, CI-linked respiration was stimulated by ADP, and inhibition of CI caused a small decrease in respiration [Figure 1F], suggesting intactness of OMM and better preservation of the electron transport chain (ETC).

Comparing respiration within the isogenic pair of cell lines, T-cam/T-cam Cis showed significant (-35%) inhibition of routine (ce) respiration. The inhibition of CI-linked OXPHOS capacity - with parameters 2D (-53%), 2D; c (-50%), 3G (-49%), CI&II OXPHOS and ET capacity - 4S (-51%) and 5U (-38%), and CII-linked ET capacity - 6Rot (-40%) in the resistant T-cam Cis line suggests inhibition of both CI-linked and CII-linked pathways. Addition of Gp compensated for the decline in the CII-linked pathway, as the ET capacity of CII&GpDH was the same as in the T-cam cell line [Figure 1G]. The difference within the H12.1/ H12.1 ODM pair was the decline in routine respiration (-36%) and CIV activity (-47%) in the resistant H12.1 ODM cell line [Figure 1E]. CIV activity also declined in the 2102EP Cis (-46%) *vs*. 2102EP line [Figure 1D]. Moreover, in the 2102 Cis line, CI&II OXPHOS and ET capacity were reduced [Figure 1D].

In summary, resistant cell lines differed from the sensitive cell lines in several ways with not uniform, but individually specific patterns. In the T-cam Cis line, CI and CII-linked pathways were negatively affected and the metabolic reprogramming increased the relative involvement of the GpDH-linked pathway in mitochondrial respiration. In H12.1 ODM and 2102EP Cis lines, CIV activity was markedly reduced. In T-cam Cis and H12.1 ODM, the defect in mitochondrial respiration negatively affected the routine respiration of intact cells. In the NTERA-2 Cis line, the OMM and ETC were more preserved compared to the NTERA-2 cell line.

### Gene expression analysis

**Figure 2:**
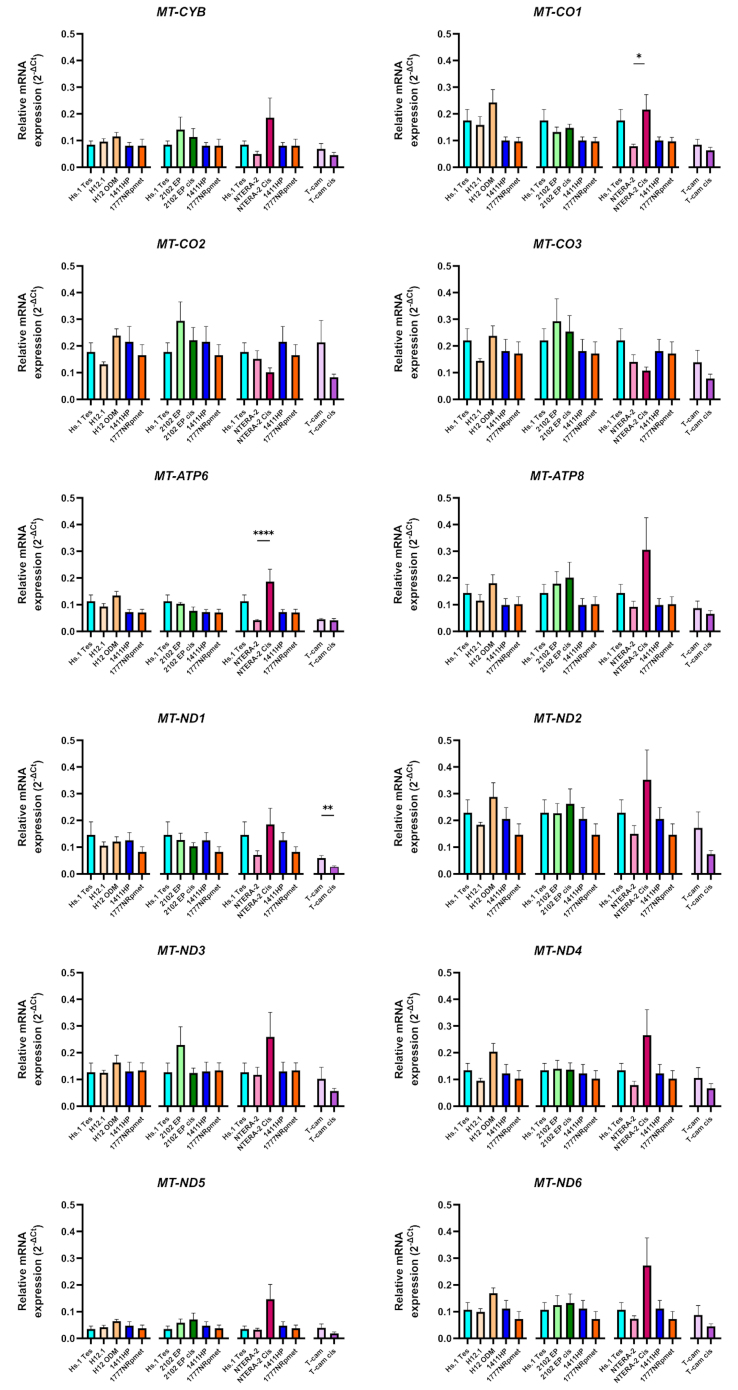
Differential gene expression of 12 mitochondrial respiratory chain genes. All CDDP-resistant (H12.1 ODM, 1411HP, 1777NRpmet, 2102EP Cis) were compared against control Hs1.Tes and each sensitive (H12.1, 2102EP and NTERA-2) TGCT cell lines. The graphical arrangement always follows a combination of the control cell line Hs.1 Tes, TGCT sensitive line and its resistant pair (H12.1/H12.1 ODM, 2102EP/2102EP Cis, NTERA-2/NTERA-2 Cis), and two metastatic resistant lines (1411HP, 1777NRpmet), which do not have their own parental cell line. The seminomatous pair T-cam/T-cam Cis was compared separately. Data are presented as mean relative mRNA expression (2-ΔCt) from three technical and three biological replicates. Error bars represent SD. ^*^P ≤ 0.05; ^**^P ≤ 0.01; ^***^P ≤ 0.001. TGCT: Testicular germ cell tumors; CDDP: cisplatin; Hs1.Tes: control cell line; 1411HP, 1777NRpmet: intrinsically resistant TGCT cell lines; H12.1/H12.1ODM, 2102EP/2102EP Cis, NTERA-2/NTERA-2 Cis: non-seminomatous TGCT cell line pairs (sensitive/resistant).

In parallel with mitochondrial respiration activity, we evaluated the expression level of mitochondrial genes encoding the respiratory enzymes [Figure 2]. Analysis of the expression of respiratory genes showed that all mitochondrially encoded respiratory genes were highly expressed and did not show any statistically significant differences among TGCT cell lines. The only exception was observed when NTERA-2/NTERA-2 Cis lines were compared, showing significant upregulation of *MT-CO1* and *MT-ATP6* (*P* < 0.05 and *P* < 0.001) gene expression in the resistant cell line. Similarly, a comparison of T-cam/T-cam Cis showed significant downregulation of *MT-ND1* (*P* < 0.01) gene expression in the resistant cell line [Figure 2] compared to its sensitive counterpart. However, this expression may not fully reflect the expression (and function) of the given proteins, as the expression of nuclear-encoded respiratory genes was not evaluated in this study.

### Analysis of metabolic pathway dependence

**Figure 3:**
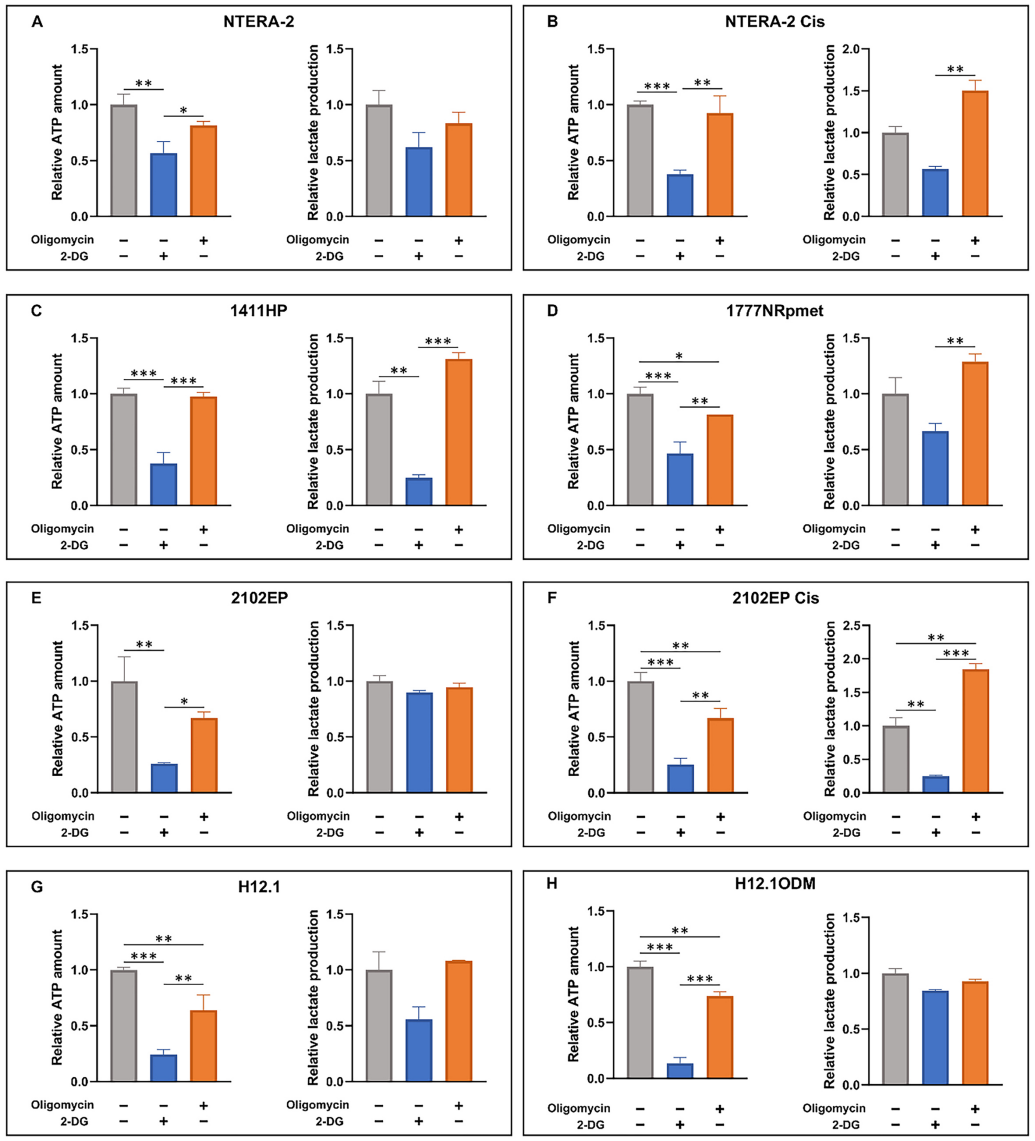
Comparison of metabolic pathway dependence of TGCT cell lines. TGCT cell lines NTERA-2 (A) and NTERA-2 Cis (B), 1411HP (C), 1777NRpmet (D), 2102EP (E), 2102EP Cis (F), and H12.1 (G), H12.1 ODM (H) were treated with combination of two inhibitors, 2-DG inhibitor to inhibit glycolysis and oligomycin to inhibit the OXPHOS metabolic pathway. Absorbance (450 nm) and luminescence values were normalized to untreated TGCT cells. To determine the metabolic pathway dependence of individual TGCT cell lines, we compared changes in ATP and lactate production after the application of inhibitors. Data are presented as mean relative ATP and lactate amounts from three technical and three biological replicates. Error bars represent SD. ^*^P ≤ 0.05; ^**^P ≤ 0.01; ^***^P ≤ 0.001. TGCT: Testicular germ cell tumors; OXPHOS: oxidative phosphorylation.

In cancer cells, numerous mutations in mtDNA, as well as changes in the expression of respiratory genes, can affect mitochondrial metabolic pathways and ATP production. Accordingly, these mutations can lead to the progression of the disease or contribute to chemoresistance development. In order to reveal a possible association of the identified mt genome mutations with the dependence of individual cell lines on a metabolic pathway or a metabolic shift, we next focused on the analysis of the preferential use of glycolysis or OXPHOS for ATP generation by TGCT cells. Using 2-DG and oligomycin inhibitors, we determined the concentration of intracellular ATP and the amount of lactate released from the cells. This portrayed the dynamics of intracellular metabolic activity and enabled the evaluation of the corresponding metabolic shift. In the sensitive NTERA-2 cell line, ATP levels were reduced by the administration of both inhibitors, 2-DG and oligomycin. The amount of lactate production was reduced by oligomycin [Figure 3A], indicating that the metabolism of the NTERA-2 cell line is dependent on OXPHOS. The opposite trend was observed in resistant cell line NTERA-2 Cis, where ATP levels were significantly reduced by inhibition of the glycolytic system, but remained relatively unchanged by oligomycin treatment. The amount of lactate production was increased by oligomycin treatment [Figure 3B], indicating that the metabolism of this cell line is highly dependent on glycolysis. Similarly, the metastatic cell line 1411HP showed decreased ATP level only after 2-DG treatment. The amount of lactate production was reduced by 2-DG treatment but increased by oligomycin treatment [Figure 3C]. This suggests that this cell line is also dependent on the glycolytic pathway. In the case of the metastatic cell line, 1777NRpmet, we observe reduced ATP levels when treated with both inhibitors, but lactate levels were reduced after 2-DG and increased after oligomycin treatment [Figure 3D]. We, therefore, hypothesize that this cell line might depend on, or equally utilize, both pathways.

In other TGCT cell line pairs, 2102EP/ 2102EP Cis and H12.1/ H12.1ODM, ATP levels were reduced by treatment with both inhibitors. In addition, we observed a small reduction of lactate production in 2102EP [Figure 3E], but the lactate production increased in 2102EP Cis [Figure 3F] and H12.1 TGCT cell lines [Figure 3G] after inhibition of ATP synthesis by oligomycin, indicating activation of the glycolytic pathway. Reduction of lactate production was also observed in H12.1ODM TGCT cell lines [Figure 3H]. We assume that 2102EP and H12.1 ODM rely more on OXPHOS, and H12.1 and 2102EP Cis more on the glycolytic pathway.

## DISCUSSION

TGCTs have typically been characterized by frequent chromosomal abnormalities, but low rate of somatic mutations. However, the development of advanced NGS technologies has enabled the identification of new mutations, variants, and alterations in nuclear DNA (nDNA) and mtDNA in many malignancies, including TGCTs^[4]^. To date, Genome-Wide Association Studies (GWAS) have identified more than 30 risk loci for TGCTs, suggesting that a polygenic model better reflects the genetic environment of the disease^[18]^. The vast majority of post-pubertal TGCTs have increased copy number variations (CNVs) of whole or partial segments of the short arm (p) of chromosome 12, manifested as isochromosome 12p [or “I (12p)”], but genomic features contributing to the onset and progression or chemosensitive or resistant phenotype in TGCTs remain unknown^[19]^.

CDDP therapeutically targets both nDNA and mtDNA. We hypothesized that despite the absence of functional causal mutations in TGCTs, their tumorigenicity and resistance may be mediated by a number of yet undescribed genetic changes, specifically in the mt genome. In the mtDNA of cancer cells, numerous mutations have been found. These do not inactivate mitochondrial functions, but rather change the energy metabolism to support the growth of cancer cells. Altered mtDNA profiles, including CNVs and mutations, modify mitochondrial bioenergetics and impair mitochondrial-nuclear signaling, which accelerates tumorigenesis^[20]^. In addition, the accumulation of mitochondrial mutations relates to the progression of the disease, as well as the origin and development of therapy resistance^[21]^.

Analyzing the mutational status of the mt genome of individual TGCT cell lines, we identified new mutations, most of them yet undescribed, and revealed their unique representation in resistant TGCT cell lines. Despite the fact that the highest mutation frequency was observed in *MT-TP*, *-TR*, -*TQ* genes (mitochondrially encoded tRNA proline, tRNA arginine, tRNA glutamine), these mutations were distributed equally in all cell lines and were not found specific in any TGCT cell line [Table 4]. However, new mutations were identified in the genes encoding mitochondrial subunits of the respiratory chain: *MT-ND, MT-CO1-3, MT-ATP6, MT-CYB* genes, and *MT-RNR* encoding mt 12S rRNA. As listed in Table 2, *MT-ND1* mutations were identified in resistant H12.1ODM and metastatic 1411HP cell lines, *MT-ND2* mutation in the 2102EP Cis line, *MT-ND3* and *MT-ND5* mutations in the 1411HP cell line. In T-cam Cis, mutations in *MT-ND3* and *MT-ND5* genes, and in the H12.1ODM cell line, *MT-ND5* mutation were detected. However, none of these mutations were detected in the resistant 1777NRpmet cell line. On the other hand, in metastatic 1777NRpmet, mutations in *MT-RNR1, MT-RNR2, MT-CO1,* and *MT-CO3* were detected but, vice versa, none of these mutations was identified in any other TGCT cell line. We assume that these SNP variations can potentially be associated with CDDP resistance. These mutations need to be verified and validated in a cohort of TGCT patients. To date, no such data are available for TGCTs, neither in The Cancer Genome Atlas nor in any similar databases.

Contribution of the new mutations in respiratory genes to the drug resistance identified in resistant TGCT cells remains unclear. However, most of the identified mt gene variations, mutations and/or SNPs in given positions have been associated with neurodegenerative, autoimmune diseases, or other malignancies^[22-24]^. For example, the homoplasmic variant 3481G > A located in the *MT-ND1* gene is a known pathological variant associated with MELAS disease (mitochondrial encephalomyopathy with lactic acidosis and stroke-like episodes), progressive encephalomyopathy, or Leigh syndrome. This mutation causes a glutamate (G) to lysine (E59K) change that affects a highly conserved residue in the first side loop of the ND1 subunit matrix^[25,26]^. Variants in genes *MT-ND1 421*6T > C, *MT-ND5* 12633C > A and 13368G > A, *MT-ND4* 11251A > G and 11719G > A, *MT-CO1* 7028C > T, *MT-CYB* 15452C > A and 14766C > T, *MT-RNR2* 1888G > A, *MT-ATP6* 8856G > A and *MT-ND2* 4917A > G were described as secondary LHON mutations in Parkinson’ disease, Leight syndrome, or Leber’s hereditary optic neuropathy^[27-29]^. Similarly, mutations in *MT-CR* gene 73A > G were found accumulated in the brains of patients with Alzheimer’s disease^[30]^. Mutations in *MT-ND5* 12633C > A and *MT-ND4* 11719G > A genes were found in patients with multiple sclerosis^[31]^. On the other hand, several of these mutations, including those we identified, are present in various malignancies. Polymorphism in *MT-ND1* gene 4216T > C was found in thyroid tumor patients. Differential distributions of mtDNA sequence variants between carcinomas and healthy controls were identified frequently in the genes encoding CI of mitochondrial ETC. The authors of this study proposed that somatic mtDNA mutations might be involved in thyroid tumorigenesis and disease progression^[32]^. Similarly, A/G transition in nucleotide position 4216 of the *MT-ND1* gene was observed in acute leukemia induced by clonal myeloid disorders^[33]^. Mutations in the somatic variant of mtDNA in the *MT-RNR1* gene, 709G > A and *MT-CR*, 195T > C were also observed in patients with ovarian and prostate cancer, glioblastomas, noninvasive thyroid follicular neoplasms, and nasopharyngeal tumors^[34-37]^. Mutations in *MT-CYB* variation 15607A > G were present in breast cancer patients but were insignificant compared to healthy tissue^[38]^. Mutations in *MT-TP* gene 1603T > C and 16519T > C were found in patients with nasopharyngeal carcinoma and ovarian cancer^[37]^. Somatic mtDNA mutation 8697G > A of the *MT-ATP6* gene was observed in Hürthle cell adenoma (HCA) and Hürthle cell follicular carcinoma (HCFC)^[39]^. Lastly, *MT-TR* gene variant 10463T > C was found in endometrial tumor samples^[40]^. Abril *et al*. demonstrated decreased expression levels of *MT-RNR, MT-CO2, and MT-ATP6* in prostate cancer^[41]^. Another study indicated that *MT-RNR1* could be a predictor of ovarian cancer chemotherapy outcome^[42]^. Furthermore, the polymorphism of this gene, *MT-RNR1* G709A, has been associated with poor prognosis and is considered an important risk factor in patients with hepatocellular carcinoma^[43]^. Ghaffarpour *et al*. reported that the mitochondrially encoded subunit of *MT-ATP6* was more susceptible to mutations leading to the induction of metabolic imbalance in breast cancer patients^[44]^. Pathogenic mutations in *MT-ND* genes have been found to increase tumor invasiveness and the ability to metastasize. Koshikawa *et al*. demonstrated that single nucleotide variants (SNVs) in *MT-ND* genes, with the potential to reduce respiratory chain CI activity, are involved in the spread of distant metastases in non-small cell lung cancer (NSCLC) and colon cancer^[22]^. In line with this observation, this is the first time that mutations in *MT-ND* genes have been identified in TGCT cell lines and reported in association with resistant phenotype and/or metastatic potential. The SNP variations identified in our study have been described in various other malignancies, including those with worse treatment outcomes, which further supports their potential in CDDP resistance development.

To reveal the potential functional consequence of identified mt genome changes, we focused on the comparison of relative expression of mitochondria-encoded respiratory genes, assessment of respiration activity of individual TGCT cell lines and their metabolic pathway dependence for ATP production. The most significant differences were observed in 1777NRpmet in comparison to the 1411HP TGCT cell line.

Although both lines represent resistant metastatic TGCT cells, their mitochondrial respiration was completely opposite; CI-linked OXPHOS capacity (after ADP addition - the respiration associated with ATP production) was significantly decreased in 1411HP and significantly increased in 1777NRpmet. The resistant TGCT cell line 1777NRpmet showed the highest O_2_ consumption after the addition of individual substrates. On the other hand, resistant cell line 1411HP did not respond to ADP. The respiration increased slightly after the addition of cyt c, suggesting impaired integrity of the OMM. There was almost no further response to any of the added substrates or uncoupler. CI inhibitor caused a slight decline in respiration in the 1411HP cell line.

We hypothesize that different respiratory chain performances may relate to the newly identified mutations in respiratory genes. In the 1777NRpmet cell line, these were mainly in the *MT-CO* genes (CIV), while in the 1411HP cell line, we identified mutations mainly in the *MT-ND* genes (CI). In addition, 1411HP was the only cell line with mutations in the *MT-ATP6* gene (CV), which is important for the formation of ATP in the respiratory chain. In the 1411HP cell line, SNV 12425 - > A in the *ND5* gene caused a frameshift with potential pathological effects, which may affect the activity of CI and the initial conversion of incoming electrons to the respiratory system. Another mutation, 3902 ACCTT > AAGGT in the *MT-ND1* gene, caused an inversion of seven nucleotides located between positions 3902 and 3908. This may be a potential pathological variant, as the mutation converts three consecutive amino acids from Asp-Leu-Ala at amino acid positions 199-201 to Gly-Lys-Val in a highly conserved region of the *MT-ND1* gene. Replacing negatively charged Asp199Gly and hydrophobic Leu-200 with a basic Lys residue can be particularly detrimental^[45]^. Despite these observations, the level of gene expression of *MT-ATP6, MT-ND3*, and *MT-ND5* in 1411HP was unaffected compared to the sensitive counterpart.

In the 1777NRpmet cell line, the O_2_ consumption was the highest, but none of the mutations found in 1411HP were detected in this cell line. The gene expression of all mitochondrial respiratory chain genes in 1411HP and 1777NRpmet cells was almost identical [Figure 2]. However, the analysis of metabolic pathway dependence showed that resistant metastatic 1411HP preferred glycolysis while 1777NRpmet cells utilized both OXPHOS and glycolysis for ATP production [Figure 3D].

The statistically significant differences in gene expression of the respiratory genes were recorded only in NTERA-2 Cis and T-cam Cis cell lines. Resistant NTERA-2 Cis expressed more *MT-CO1* and *MT-ATP6* genes compared to the sensitive counterpart NTERA-2. The expression of all *MT-ND* genes, *MT-CYB*, and *MT-ATP8* was also upregulated. The increased expression of the *MT-ATP6* gene can be associated with a better response to ADP. However, O_2_ consumption of the NTERA-2 Cis line was low and almost identical to the sensitive NTERA-2 parental cell line [Figure 1F], and these resistant cells preferentially utilized glycolysis for ATP production [Figure 3B]. In the T-cam Cis cell line, the expression of the *MT-ND1* gene was significantly downregulated compared to the parental sensitive T-cam cell line, potentially explaining the reduced function of the CI [Figure 1G]. The same pattern of expression downregulation was observed in all other respiratory genes. The analysis of respiratory chain function revealed significantly lower O_2_ consumption. In T-cam Cis, mutations in *MT-ND3* and *MT-ND5* genes and significantly lower *MT-ND1* expression can be associated with the decline in CI-linked respiration.

In the resistant H12.1ODM cell line, all mitochondrial respiratory chain genes showed a tendency of the expression upregulation [Figure 2], but surprisingly, respiration and O_2_ consumption parameters were similar to its sensitive counterpart H12.1 cell line [Figure 1E]. The phenomenon of increased gene expression with no effect on respiration was also observed in the isogenic pair of cell lines NTERA-2/NTERA-2 Cis [Figure 1F]. In the H12.1 ODM, the mutations in *MT-CO2* and *MT-CYB* genes could cause a decline in CIV activity. Nevertheless, resistant H12.1ODM cells preferentially utilized OXPHOS, which could potentially be explained by respiratory genes’ increased expression [Figure 3H].

When comparing resistant 2102EP Cis *vs.* sensitive 2102EP cell lines, no differences were observed in gene expression of respiratory genes [Figure 2], but the respiration and O_2_ consumption were significantly decreased in the resistant 2102EPCis line [Figure 1D]. Similar to 1411HP and T-cam Cis, 2102EP Cis cells manifested significantly reduced respiration without any essential difference in the expression of respiratory genes, and for ATP production, they also preferentially utilized glycolysis [Figure 3F].

Due to bi-directional communication between the nuclear and mt genomes, a wide range of mitochondrial disorders causing clinical syndromes relate to altered OXPHOS^[23]^. Several studies have reported changes in respiratory chain genes contributing to the induction of tumorigenesis^[24,46-49]^. Concordantly, analysis within TGCT cell lines identified high mutation frequency of all respiratory chain genes [Table 4]. The mutational frequency helps to prioritize the genes and pathways for potential therapeutical targeting^[50]^.

Cancer cells are known to produce most of their ATP through glycolysis, even under aerobic conditions^[51]^. Consequently, a correlation between glycolytic ATP production and tumor cell aggressiveness has been revealed^[24]^. The concept of aerobic glycolysis being a universal property of malignant cells has been further challenged and it has been shown that mitochondria in tumor cells do respire and produce ATP^[52]^. It was shown that despite the high rates of glycolysis, many tumors or subpopulations of tumor cells also rely on OXPHOS^[53]^. An increased dependence of tumor cells on OXPHOS was also observed in the advanced stages of the malignant disease^[54]^. The ability to switch metabolism during tumorigenesis reflects the capacity of mitochondria to adapt to the metabolic demands of cancer cells, ensuring their aberrant survival^[55]^. In regard to preferential utilization of metabolic pathways used for ATP production, our results of the measurements of ATP and lactate levels showed that sensitive cell lines NTERA-2 and 2102EP preferred OXPHOS while glycolysis was typical for resistant NTERA-2 Cis, 2102EP Cis, and 1411HP cell lines. Metastatic 1777NRpmet cells seem to utilize both OXPHOS and glycolysis, so the dependence on a specific energy pathway is, therefore, not fully clear for this TGCT cell line. We hypothesize that this metastatic resistant cell line might be more susceptible to metabolic switching in response to the tumor microenvironment, such as intermittent hypoxia. An isogenic pair of cell lines H12.1 and H12.1ODM showed the opposite dependence, sensitive H12.1 preferring glycolysis, while resistant H12.1ODM OXPHOS. This divergence could potentially be explained by different ways of generation of resistant phenotype, being derived from its sensitive parental H12.1 cell line by cultivation in differentiation-inducing media.

Due to the close proximity of mtDNA to the ETC, the level of oxidatively modified bases in mtDNA is 10-20 times higher than in nDNA. The resulting oxidative damage can lead to lethal loss of ET and ATP generation, leading to cellular death. Together with oxidized proteins and induced lipid peroxidation, ROS compromise the protective properties of biological membranes^[56]^. ROS-induced oxidative damage is likely a major source of mitochondrial genomic instability leading to respiratory dysfunction^[5]^ and consequent apoptosis. Accordingly, higher OXPHOS in sensitive TGCT cell lines could increase the production of harmful ROS that leads to significant mitochondrial damage, triggering apoptosis in sensitive cells. On the other hand, in resistant TGCT cells, preference for glycolysis and reduction in OXPHOS decreases ROS to levels below the required threshold needed to trigger apoptosis. Exceptionally, advanced stages potentially utilize both glycolysis and OXPHOS. Consequently, resistant cells cumulate all damages caused by ROS or therapeutics, survive, adapt, and progress, further increasing their resistance and aggressiveness.

Divergent behavior of resistant metastatic 1411HP and 1777NRpmet and opposite in paired H12.1/ H12.1ODM cell lines confirms histological variability of TGCTs and potentially reflects different ways of resistant phenotype acquisition. A detailed understanding of these processes provides the basis for potential novel diagnostics and therapeutic targeting of mitochondria in cancer treatment^[57]^.

Summarizing our data, we conclude that TGCT cell lines with different degrees of CDDP resistance showed significant differences in all our analyses. While TGCTs that respond better to CDDP tended to prefer OXPHOS, resistant TGCTs relied more on glycolysis to supplement the ATP requirements of cancer cells. The resistant metastatic 1777NRpmet TGCT cell line showed the highest respiration, but also an exceptional manner of utilizing both OXPHOS and glycolysis. The respiratory gene expression was moderately changed in selected resistant cell lines, but could partially explain different respiration capacities and preferences of metabolic pathways. Analysis of genetic variability identified panels of mutations that can distinguish individual sensitive or resistant phenotypes. Despite the need for disclosure of whether these features are the cause or consequence of CDDP resistance and advanced tumor aggressiveness, these findings provide solid evidence that changes in the mt genome and metabolism might lead to mitochondrial dysfunction, potentially providing a growth advantage to cancer cells. Identified mutations in mt genes associated with different mitochondrial respiration parameters, gene expression patterns, and preferences for metabolic pathways provide potential novel molecular biomarkers that can recognize resistant TGCT phenotype or specify its histological classification. Similar to other studies based on cell line models, our study carries certain limitations associated with possible clonal selection, incomplete identification of mutations, limited heterogeneity, or artefacts introduced during the process of cell culturing that can result in an inaccurate reflection of the primary tumor tissue. The complexity of this study is also limited by investigating genetic mutations and alterations only within mtDNA-encoded mitochondrial genes without providing parallel information about nuclear-encoded mitochondrial genes. Therefore, our observations require further validation on a cohort of samples from TGCT patients to be confirmed as relevant and valid for molecular diagnostics of chemoresistance. Accordingly, precise patient stratification and tailored personalized therapeutical settings for resistant TGCT patients can be improved.

## DECLARATIONS

### Availability of data and materials

All the data and materials used in our study are available from the corresponding author upon reasonable request.

### Copyright

© The Author(s) 2024.

## References

[1] Ottaviano M, Giunta EF, Rescigno P (2021). The enigmatic role of TP53 in germ cell tumours: are we missing something?. Int J Mol Sci.

[2] Adra N, Einhorn LH (2017). Testicular cancer update. Clin Adv Hematol Oncol.

[3] Romano FJ, Rossetti S, Conteduca V (2016). Role of DNA repair machinery and p53 in the testicular germ cell cancer: a review. Oncotarget.

[4] Zhang CJ, Li ZT, Shen KJ, Chen L, Xu DF, Gao Y (2021). Characterization of progression-related alternative splicing events in testicular germ cell tumors. Asian J Androl.

[5] Gogvadze V, Orrenius S, Zhivotovsky B (2008). Mitochondria in cancer cells: what is so special about them?. Trends Cell Biol.

[6] Ma K, Chen G, Li W, Kepp O, Zhu Y, Chen Q (2020). Mitophagy, mitochondrial homeostasis, and cell fate. Front Cell Dev Biol.

[7] Protasoni M, Zeviani M (2021). Mitochondrial structure and bioenergetics in normal and disease conditions. Int J Mol Sci.

[8] Bestwick ML, Shadel GS (2013). Accessorizing the human mitochondrial transcription machinery. Trends Biochem Sci.

[9] Liu W, Wang Y, Luo J, Yuan H, Luo Z (2019). Genetic polymorphisms and platinum-based chemotherapy-induced toxicities in patients with lung cancer: a systematic review and meta-analysis. Front Oncol.

[10] Gammage PA, Frezza C (2019). Mitochondrial DNA: the overlooked oncogenome?. BMC Biol.

[11] Guzy RD, Schumacker PT (2006). Oxygen sensing by mitochondria at complex III: the paradox of increased reactive oxygen species during hypoxia. Exp Physiol.

[12] Roška J, Wachsmannová L, Hurbanová L (2020). Differential gene expression in cisplatin-resistant and -sensitive testicular germ cell tumor cell lines. Oncotarget.

[13] Mueller T, Mueller LP, Luetzkendorf J, Voigt W, Simon H, Schmoll HJ (2006). Loss of Oct-3/4 expression in embryonal carcinoma cells is associated with induction of cisplatin resistance. Tumour Biol.

[14] Balin SJ, Cascalho M (2010). The rate of mutation of a single gene. Nucleic Acids Res.

[15] Pesta D, Gnaiger E (2012). High-resolution respirometry: OXPHOS protocols for human cells and permeabilized fibers from small biopsies of human muscle. Methods Mol Biol.

[16] https://wiki.oroboros.at/index.php/SUIT-008_O2_ce-pce_D025.

[17] https://wiki.oroboros.at/images/archive/4/40/20200417143232%21MiPNet17.04_CitrateSynthase.pdf.

[18] Batool A, Karimi N, Wu XN, Chen SR, Liu YX (2019). Testicular germ cell tumor: a comprehensive review. Cell Mol Life Sci.

[19] Howitt BE, Berney DM (2015). Tumors of the testis: morphologic features and molecular alterations. Surg Pathol Clin.

[20] Marullo R, Werner E, Degtyareva N (2013). Cisplatin induces a mitochondrial-ROS response that contributes to cytotoxicity depending on mitochondrial redox status and bioenergetic functions. PLoS One.

[21] Taylor-Weiner A, Zack T, O'Donnell E (2016). Genomic evolution and chemoresistance in germ-cell tumours. Nature.

[22] Koshikawa N, Akimoto M, Hayashi JI, Nagase H, Takenaga K (2017). Association of predicted pathogenic mutations in mitochondrial ND genes with distant metastasis in NSCLC and colon cancer. Sci Rep.

[23] Zeviani M, Di Donato S (2004). Mitochondrial disorders. Brain.

[24] Simonnet H, Alazard N, Pfeiffer K (2002). Low mitochondrial respiratory chain content correlates with tumor aggressiveness in renal cell carcinoma. Carcinogenesis.

[25] Malfatti E, Bugiani M, Invernizzi F (2007). Novel mutations of ND genes in complex I deficiency associated with mitochondrial encephalopathy. Brain.

[26] Moslemi AR, Darin N, Tulinius M, Wiklund LM, Holme E, Oldfors A (2008). Progressive encephalopathy and complex I deficiency associated with mutations in MTND1. Neuropediatrics.

[27] Kösel S, Grasbon-Frodl EM, Mautsch U (1998). Novel mutations of mitochondrial complex I in pathologically proven Parkinson disease. Neurogenetics.

[28] Vives-Bauza C, Andreu AL, Manfredi G (2002). Sequence analysis of the entire mitochondrial genome in Parkinson’s disease. Biochem Biophys Res Commun.

[29] van der Walt JM, Nicodemus KK, Martin ER (2003). Mitochondrial polymorphisms significantly reduce the risk of Parkinson disease. Am J Hum Genet.

[30] Coskun PE, Beal MF, Wallace DC (2004). Alzheimer’s brains harbor somatic mtDNA control-region mutations that suppress mitochondrial transcription and replication. Proc Natl Acad Sci U S A.

[31] Alharbi MA, Al-Kafaji G, Khalaf NB (2019). Four novel mutations in the mitochondrial *ND4* gene of complex I in patients with multiple sclerosis. Biomed Rep.

[32] Yeh JJ, Lunetta KL, van Orsouw NJ (2000). Somatic mitochondrial DNA (mtDNA) mutations in papillary thyroid carcinomas and differential mtDNA sequence variants in cases with thyroid tumours. Oncogene.

[33] Linnartz B, Anglmayer R, Zanssen S (2004). Comprehensive scanning of somatic mitochondrial DNA alterations in acute leukemia developing from myelodysplastic syndromes. Cancer Res.

[34] Brandon M, Baldi P, Wallace DC (2006). Mitochondrial mutations in cancer. Oncogene.

[35] Kirches E, Krause G, Warich-Kirches M (2001). High frequency of mitochondrial DNA mutations in glioblastoma multiforme identified by direct sequence comparison to blood samples. Int J Cancer.

[36] Xu B, Reznik E, Tuttle RM (2019). Outcome and molecular characteristics of non-invasive encapsulated follicular variant of papillary thyroid carcinoma with oncocytic features. Endocrine.

[37] Pang LJ, Shao JY, Liang XM, Xia YF, Zeng YX (2008). Mitochondrial DNA somatic mutations are frequent in nasopharyngeal carcinoma. Cancer Biol Ther.

[38] Mbaye F, Dem A, Fall M (2014). Genetic diversity of breast cancer in senegalese women: new insight from somatic mutations. J Health Sci.

[39] Máximo V, Soares P, Lima J, Cameselle-Teijeiro J, Sobrinho-Simões M (2002). Mitochondrial DNA somatic mutations (point mutations and large deletions) and mitochondrial DNA variants in human thyroid pathology: a study with emphasis on Hürthle cell tumors. Am J Pathol.

[40] Cardaioli E, Dotti MT, Hayek G, Zappella M, Federico A (1999). Studies on mitochondrial pathogenesis of Rett syndrome: ultrastructural data from skin and muscle biopsies and mutational analysis at mtDNA nucleotides 10463 and 2835. J Submicrosc Cytol Pathol.

[41] Abril J, de Heredia ML, González L (2008). Altered expression of *12S/MT-RNR1, MT-CO2/COX2,* and *MT-ATP6* mitochondrial genes in prostate cancer. Prostate.

[42] Bragoszewski P, Kupryjanczyk J, Bartnik E, Rachinger A, Ostrowski J (2008). Limited clinical relevance of mitochondrial DNA mutation and gene expression analyses in ovarian cancer. BMC Cancer.

[43] Lin YH, Chu YD, Lim SN, Chen CW, Yeh CT, Lin WR (2021). Impact of an MT-RNR1 gene polymorphism on hepatocellular carcinoma progression and clinical characteristics. Int J Mol Sci.

[44] Ghaffarpour M, Mahdian R, Fereidooni F, Kamalidehghan B, Moazami N, Houshmand M (2014). The mitochondrial *ATPase6* gene is more susceptible to mutation than the *ATPase8* gene in breast cancer patients. Cancer Cell Int.

[45] Musumeci O, Andreu AL, Shanske S (2000). Intragenic inversion of mtDNA: a new type of pathogenic mutation in a patient with mitochondrial myopathy. Am J Hum Genet.

[46] Moindjie H, Rodrigues-Ferreira S, Nahmias C (2021). Mitochondrial metabolism in carcinogenesis and cancer therapy. Cancers.

[47] Tan AS, Baty JW, Berridge MV (2014). The role of mitochondrial electron transport in tumorigenesis and metastasis. Biochim Biophys Acta.

[48] Gasparre G, Porcelli AM, Lenaz G, Romeo G (2013). Relevance of mitochondrial genetics and metabolism in cancer development. Cold Spring Harb Perspect Biol.

[49] Badrinath N, Yoo SY (2018). Mitochondria in cancer: in the aspects of tumorigenesis and targeted therapy. Carcinogenesis.

[50] Mendiratta G, Ke E, Aziz M, Liarakos D, Tong M, Stites EC (2021). Cancer gene mutation frequencies for the U.S. population. Nat Commun.

[51] Warburg O, Wind F, Negelein E (1927). The metabolism of tumors in the body. J Gen Physiol.

[52] Weinhouse S (1976). The Warburg hypothesis fifty years later. Z Krebsforsch Klin Onkol Cancer Res Clin Oncol.

[53] Goto M, Miwa H, Shikami M (2014). Importance of glutamine metabolism in leukemia cells by energy production through TCA cycle and by redox homeostasis. Cancer Invest.

[54] Faubert B, Solmonson A, DeBerardinis RJ (2020). Metabolic reprogramming and cancer progression. Science.

[55] Cannino G, Ciscato F, Masgras I, Sánchez-Martín C, Rasola A (2018). Metabolic plasticity of tumor cell mitochondria. Front Oncol.

[56] Andreyev AY, Kushnareva YE, Starkov AA (2005). Mitochondrial metabolism of reactive oxygen species. Biochemistry.

[57] Sullivan LB, Gui DY, Hosios AM, Bush LN, Freinkman E, Vander Heiden MG (2015). Supporting aspartate biosynthesis is an essential function of respiration in proliferating cells. Cell.

